# Easy Obtainment and Biological Applicability of Pinocarvyl Acetate by Encapsulating of the *Microlicia graveolens* Essential Oil in Oil-in-Water Nanoemulsions

**DOI:** 10.3390/pharmaceutics17091130

**Published:** 2025-08-29

**Authors:** Janaína Brandão Seibert, Tatiane Roquete Amparo, Lucas Resende Dutra Sousa, Ivanildes Vasconcelos Rodrigues, Alicia Petit, Pauline Pervier, Mariana Costa Azevedo, Policarpo Ademar Sales Junior, Silvane Maria Fonseca Murta, Cláudia Martins Carneiro, Luiz Fernando de Medeiros Teixeira, Gustavo Henrique Bianco de Souza, Paula Melo de Abreu Vieira, Orlando David Henrique dos Santos

**Affiliations:** 1 Department of Entomology and Acarology, “Luiz de Queiroz” College of Agriculture, Federal University of São Paulo, Piracicaba 13418-260, Brazil; janainaseibert@usp.br; 2 Department of Pharmacy, Federal University of Ouro Preto, Ouro Preto 35402-163, Brazil; tatiane.amparo@ufop.edu.br (T.R.A.); lucas.dutra@aluno.ufop.edu.br (L.R.D.S.); a.petit@hubebi.com (A.P.); p.pervier@hubebi.com (P.P.); mariana.azevedo@aluno.ufop.edu.br (M.C.A.); guhbs@ufop.edu.br (G.H.B.d.S.); 3 Department of Pharmacy, Federal University of Juiz de Fora, Governador Valadares 36036-900, Brazil; ivanildes.rodrigues@ufjf.br; 4 Institute René Rachou (Fiocruz Minas), Belo Horizonte 30190-002, Brazil; policarpo.junior@fiocruz.br (P.A.S.J.); silvane.murta@fiocruz.br (S.M.F.M.); 5 Department of Clinical Analyzes, Federal University of Ouro Preto, Ouro Preto 35402-163, Brazil; carneirocm@ufop.edu.br (C.M.C.); lfmt@ufop.edu.br (L.F.d.M.T.); 6 Department of Biological Sciences, Federal University of Ouro Preto, Ouro Preto 35402-136, Brazil; paula@ufop.edu.br

**Keywords:** *Microlicia graveolens*, essential oil, *cis*-pinocarvyl acetate, colloidal system, nanoemulsion, antimicrobial activity

## Abstract

**Background/Objectives:** The study of biological activity of plants and their metabolites is an important approach for the discovery of new active material. However, little is known of the properties of the *Microlicia* genus. In addition to natural products, nanotechnology demonstrates considerable potential in pharmacotherapy. The utilization of nanoemulsions holds considerable promise in enhancing the efficacy of drugs, reducing dose, and therefore, lowering of toxic effects. **Methods:** In this context, antimicrobial and trypanocidal activities were evaluated to the free and encapsulated essential oil from *M. graveolens* in oil-in-water (o/w) nanoemulsion. **Results:** This oil is composed mainly of *cis*-pinocarvyl acetate (~80.0%). The nanoemulsions were prepared by phase inversion method and showed mean particle size of 58 nm, polydispercity index of 0.09, pH 7.8, zeta potential of −21.9 mV, electrical conductivity of 0.38 mS/cm, and good stability. The essential oil was active against all five Gram-positive bacteria tested, and the formulation enhanced this ability. The cytotoxicity effect on L929 cells was also reduced after encapsulation of this oil in o/w nanoemulsion. In addition, the oil and the nanoemulsion were able to inhibit the growth of *Trypanosoma cruzi*. **Conclusions:** Thus, the development of a nanoemulsion loaded with *M. graveolens* essential oil is an easy and low-cost way to obtain and deliver the *cis*-pinocarvyl acetate compound as well as allow its use in the treatment of diseases caused mainly by the genus *Listeria* and *Staphylococcus*.

## 1. Introduction

Plants represent a rich source of chemical diversity with extremely high potential to produce new therapies. Essential oils and their constituents have a wide spectrum of pharmacological activity [1]. In addition, most monoterpenes show low toxicity to mammals, being considered as safe by the United States Food and Drug Administration (USFDA) [2].

The genus *Microlicia* is native to Brazil and has approximately 170 species [3,4]. This genus remains under-explored, with recent discoveries of new species. For instance, in 2023, Rogerio and his collaborators described three novel species (*M. arenaria*, *M. dentisepala*, and *M. membranacea*) that were discovered in Minas Gerais, Brazil [5]. Pacifico and collaborators (2020) also described new species from the same region. The following species are recognized: *Microlicia capitata*, *M. coriacea*, *M. mutabilis*, *M. piranii*, *M. polychaeta*, *M. repanda*, and *M. sparsifolia* [6].

Most extant studies on species of the genus *Microlicia* are botanical descriptions, with only a limited number addressing biological properties. The dichloromethane extract of *M. crenulate* exhibited antiprotozoal activity against chloroquine-resistant *Plasmodium falciparum* [7]. The hexane extract of *M. hatschbachii* and the essential oil of *M. crenulata* exhibited antibacterial properties, thereby impeding bacterial growth [8,9].

However, there are no references to research on biological properties of *M. graveolens*, which is the focus of the present work. *M. graveolens* DC is mainly used for ornamentation and the chemical characterization of its essential oil showed high concentrations of pinocarvyl acetate [3]. This compound contributes to the characteristic woody odor of the plant [10].

Despite the various uses to combat diseases, essential oils have some physical and chemical limitations related to their hydrophobicity, volatility, and reactivity. Therefore, natural products encapsulated in nanostructured systems have been investigated as an alternative to improve the stability and effectiveness of these substances through dose reduction and therefore, lowering of toxic effects [11].

Several studies have proved the increase in the biological potential of some natural products due to use of nanostructured technologies [12,13,14]. In addition, in a review by El Asbahani et al. [15], different applications of nanoparticles loaded with essential oil have been described such as potent larvicide, antimicrobial, antioxidant, insecticide, repellent, and food preservation agents. Among drug delivery systems, there is great interest in the development of nanoemulsions due to their greater physical stability than conventional emulsions, as well as their biodegradability, biocompatibility, and easiness of preparation [16]. Nanoemulsions are colloidal dispersions consisting of nanoscale droplets stabilized by surfactants [17]. In the case of oil-in-water (o/w) nanoemulsions, nano-sized oil droplets are dispersed throughout an aqueous (water-based) phase. These droplets are surrounded by a layer of surfactants or emulsifiers that reduce interfacial tension and prevent droplet coalescence, thereby stabilizing the system [18]. The core of these droplets is lipophilic (oil-loving), rendering them optimal for encapsulating and delivering hydrophobic (water-insoluble) compounds, including essential oils [19].

Nanoemulsions have been demonstrated to be promising delivery vehicles for essential oils due to their advantageous characteristics, including their small size, high solubilization capacity, excellent encapsulation efficiency, and controlled release properties [20]. Notwithstanding the evident benefits and extensive utilization of nanoemulsions in the delivery of essential oils, there have been no reported formulation employing *M. graveolens*.

In this context, and in line with the few ethnomedicinal, phytochemical and pharmacological studies existing in the *Microlicia* genus, the essential oil of *M. graveolens* was chemically characterized and its bactericidal, fungicidal, and trypanocidal potentials were evaluated for the first time. In addition, a nanostructured system was developed and characterized to enable and enhance the biological effect of this natural product.

## 2. Materials and Methods

### 2.1. Plant Specimens and Essential Oil Extraction

*M. graveolens* was collected in Ouro Preto, Brazil (20°22′36″ S; 43°29′20″ W) in two different periods: March (1) and June (2). Voucher specimens (OUPR 29335) have been deposited at the Herbarium Prof. José Badini, Universidade Federal de Ouro Preto, Brazil and the access was registered at the Brazilian National System of Genetic Resource Management and Associated Traditional Knowledge (A4AA3E9).

The aerial parts of the plant species were subjected to hydrodistillation (Linax, Ribeirão Preto, SP-Brazil) process and the essential oil obtained was chemically characterized by GC-MS (model QP2010, Shimadzu, Kyoto, Japan) according to conditions described by Seibert et al. [21]. Calculation of Kovats index (KI) and comparison of mass spectra with reported data were performed to identify the constituents.

### 2.2. Development and Characterization of Nanoemulsion

#### 2.2.1. Preparation of Oil-in-Water Nanoemulsion

The nanoemulsion was produced by phase inversion emulsification method (PIE) as previously reported by Seibert et al. [22]. The formulation was composed of Sorbitan oleate (Span 80—Croda, Campinas, Brazil) (3.0% *w*/*v*); Polysorbate 80 (Tween 80—Synth, São Paulo, Brazil) (7.0% *w*/*v*); corn oil 100%, Mazola, Mairinque, Brazil (5.0% *w*/*v*); *M. graveolens* essential oil (5.0% *w*/*v*); and distilled water, pH 7.0, 0.8 µS/cm (80.0% *w*/*v*). Similarly, control nanoemulsion was obtained without essential oil: Sorbitan oleate (3.0% *w*/*v*), Polysorbate 80 (7.0% *w*/*v*), corn oil (10.0% *w*/*v*), and distilled water (80.0% *w*/*v*).

#### 2.2.2. Characterization of Nanoemulsion

The nanoemulsion was characterized according to mean particle size and the polydispersity index (PDI) by photon correlation spectroscopy using a Zetasizer (Zetasizer Nano model series—Nano ZS, Malvern, UK) [23]. The incidence angle of the laser in the sample cuvette was 90°. The same instrument was used to determine the zeta potential by electrophoretic mobility measurements of suspended particles and electrical conductivity measurement [23]. The pH value was determined by direct reading on a pH meter (model PH-221, Lutron, Taipei City, Taiwan). Results were expressed as the means of the three different determinations at 1, 7, and 14 days after preparation of the formulation.

#### 2.2.3. Accelerated Stability Assay

The accelerated stability assay was performed by centrifugation method [22]. The colloidal system was submitted to 3 different rotation speeds (9450; 16,800 and 26,250 rfc for 10 min) and after this process, the mean particle size and PDI were again determined to confirm the stability of the product. The analyses were performed in triplicate for different times (1, 7 and 14 days).

In order to evaluate stability under pH changes, the pH of the nanoemulsion was adjusted to 3.0, 5.0, 7.0, 9.0, and 11.0 with 0.1 mol/L HCl or 0.1 mol/L NaOH. The samples were placed at room temperature for 12 h for characterization determination of size and PDI [24].

#### 2.2.4. In Vitro Release

The in vitro drug release of nanoemulsion was analyzed by dialysis method [25]. The formulations were placed inside dialysis membrane (SnakeSkinTM Dialysis Tubing, Thermo Fisher Scientific, Rockford, IL, USA), which were then immersed in the receptor medium (ethanol 50% *v*/*v* in PBS, pH 2.5 and 7.5) under continuous stirring at 37 °C. Aliquots of 200 µL were withdrawn from receptor medium at predetermined intervals (1, 2, 4, 6, 8, 24, and 48 h) and an equal volume of fresh medium was replaced to maintain the conditions. The essential oil released was quantified by spectrophotometry at 320 nm using a Spectrophotometer—TECAN Nanoquant Infinite 200 Pro (Tecan Austria GmbH, Grodig, Austria).

To evaluate the drug-release kinetics, the following plots were generated: (1) cumulative percentage of drug release versus time to assess zero-order kinetics, (2) log cumulative drug remaining versus time for first-order kinetics, (3) cumulative percentage of drug release versus the square root of time to analyze diffusion-controlled release based on the Higuchi model; and (4) log cumulative percentage of drug release versus log time according to the Korsmeyer–Peppas model. The coefficients of determination (R2) for each model were compared to analyzing the drug-release kinetics. The formulation type, the R2 value closest to 1, and angular coefficient value (*n*) (in the case of the Korsmeyer–Peppas model) were used as parameters to determine the best kinetic profile [26].

### 2.3. Antimicrobial Assay

#### 2.3.1. Antibacterial and Antifungal Activities

In this analysis, 15 microorganisms were selected: five Gram-positive bacteria (*Staphylococcus aureus* ATCC 25923, *S. saprophyticcus* ATCC 15305, *Listeria monocytogenes* clinical isolates, *Enterococcus faecalis* ATCC 19433, and *E. faecium* ATCC 6569), seven Gram-negative bacteria (*Enterobacter aerogenes* ATCC 13048, *Salmonella typhimurium* ATCC 14028, *S. flexneri* ATCC 12022, *Escherichia coli* ATCC 25922, *Pseudomonas aeruginosa* ATCC 27853, *Providencia rettgeri* ATCC 29944, and *Klebsiella pneumoniae* ATCC 13833), and three yeasts (*Candida albicans* ATCC 14408, *C. parapsilosis* ATCC 22019, and *C. tropicalis* ATCC 750).

Initially, the screening of susceptible microorganisms to the *M. graveolens* essential oil was evaluated by agar disk diffusion method [23]. Bacteria were cultivated in Müeller–Hinton agar for 24 h at 37 °C and yeasts were cultivated in Sabouraud agar for 48 h at 37 °C. After incubation, the inoculums were obtained at 1 × 10^8^ CFU/mL (0.5 McFarland standard) and uniformly spread on agar. Blank disks (6 mm diameter) were soaked with the essential oil and control solutions. DMSO was used as negative control and tetracycline (100.0 µg/mL), moxifloxacin (100.0 µg/mL), or ketoconazole (100.0 µg/mL) were used as positive controls. The growth inhibition zone was measured, and the results were expressed as the means of the three different determinations.

In the second step, the microdilution assay was performed to compare the antimicrobial potential of the free and encapsulated essential oil by minimal inhibitory concentration (MIC) values [23]. The inoculums were prepared using only susceptible strains. In 96-well plate, microorganism (5 × 10^5^ CFU/mL) and essential oil or nanoemulsion containing the essential oil (500.0; 250.0; 125.0; 62.5; 31.2; 15.6; 7.8; 3.9; 1.9; 0.97; 0.49; 0.24; and 0.12 mg/mL) were added. Considering that the nanoemulsion formulation contained 5% of essential oil, the effective concentration of the oil ranged from 25 to 0.006 mg/mL. Negative and positive controls were used as described before and a nanoemulsion control (nanoemulsion without the essential oil) was also evaluated in parallel. The plates were incubated and then 10 µL of each sample was transferred to Petri dishes using a bacteriological loop to determine the MIC values.

#### 2.3.2. Evaluation of Trypanocidal Activity

Trypanocidal activity was evaluated using the β-galactosidase-transfected Tulahuen *T. cruzi* strain as reported previously by Romanha et al. [27]. After overnight incubation at 37 °C and 5% CO_2_, mouse L929 fibroblasts (4000) were infected with trypomastigotes (40,000) and incubated again for 48 h. The cells were treated with the essential oil or nanoemulsion in the same concentrations as the previous test. Uninfected cells (100% cure) and infected cells without treatment (0% cure) were used as negative and positive controls, respectively. In parallel, infected cells treated with Benznidazole and formulation control were used as comparative parameters. After 96 h of treatment, chlorophenol red glycoside in 0.5% Nonidet P40 was added and the microplate incubated for 18 h more. The absorbance change was measured in spectrophotometer at 570 nm. The assay was performed in triplicate, and the results were expressed as the concentration which reduced 50% of the proliferation of parasite cells (IC_50_).

### 2.4. Evaluation of Cytotoxicity

The cytotoxicity was evaluated using AlamarBlueTM assay (Thermo Fisher Scientific Rockford, IL, United States), as previously described [27]. In 96-well microplate, L929 cells (4000) were incubated for three days at 37 °C and 5% CO_2_. The cells were treated for four days with the free and encapsulated essential oil in the same concentrations as in the previous test. AlamarBlue TM was added and the plate was incubated for 4–6 h. The absorbance change was measured with a spectrophotometer at 570 and 600 nm. Untreated cells were used as negative control and formulation control was used as comparative parameters. The assay was performed in triplicate, and the results were expressed as the concentration cytotoxic for 50% of L929 cells (CC_50_).

### 2.5. Statistical Analysis

Statistical analyses were performed using GraphPad Prism^®^ 5 (GraphPad Software, San Diego, CA, USA). Friedman nonparametric test for multiple comparisons was used, followed by Dunns multiple comparison post-test for the samples, according to the samples’ normality distribution. All data are expressed as mean ± SD. A *p*-value of 0.05 or less was indicated as statistically significant.

## 3. Results

### 3.1. Extraction and Characterization of Essential Oil

The yield of oil extraction was 1.95% and 1.65% for the collection in the rainy season (1) and in the dry season (2), respectively.

The essential oils obtained from both collections were chemically characterized and the results are reported in Table 1. In the first collection (1), all constituents were identified (11 compounds) while in the second (2), 38 substances were identified, representing 98.62%. Despite the difference in the number of compounds between the collections, the percentages of compounds that are present simultaneously in both oils are 98.27% and 92.27% for collection 1 and 2, respectively.

**Figure 1:**
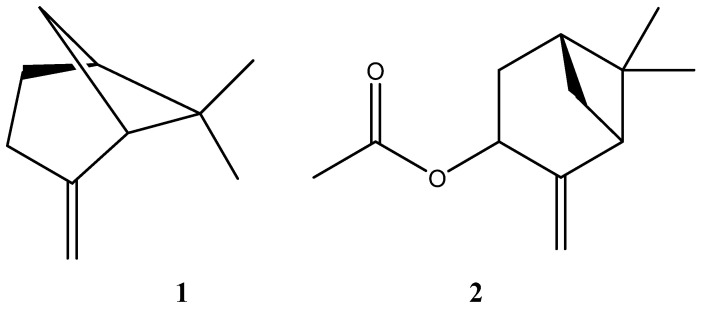
Chemical structures of β-pinene (**1**) and *cis*-pinocarvyl acetate (**2**).

In addition, both oils showed high concentrations of oxygenated monoterpene, and only collection 2 showed a small presence of hydrocarbon sesquiterpene and diterpene. In the same way, the major compounds β-pinene and *cis*-pinocarvyl acetate were found in similar proportions in collections 1 and 2, and their chemical structures are shown in Figure 1.

### 3.2. Characterization and Stability of Nanoemulsion

**Figure 2:**
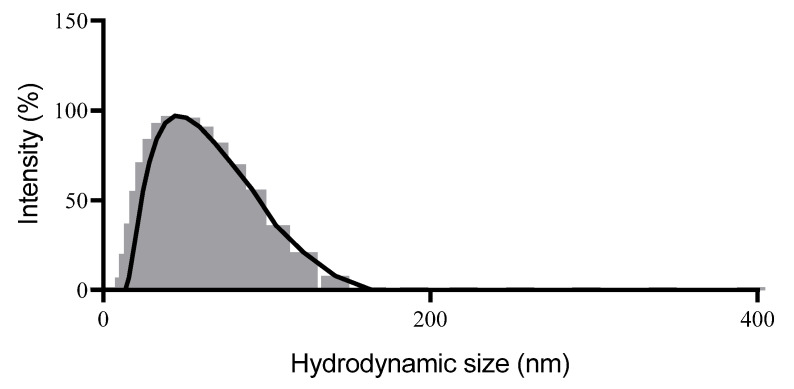
Particle size distribution histogram obtained from the Dynamic Light Scattering (DLS) of the nanoemulsion with *Microlicia graveolens* essential oil.

The mean particle size of the developed system was 58 nm with PDI of 0.09 (Figure 2).

**Figure 3:**
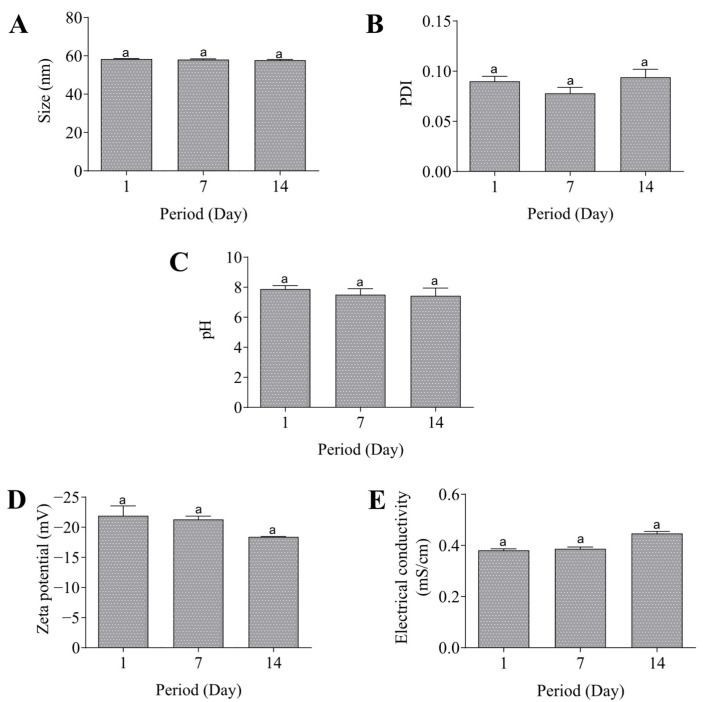
Stability of the nanoemulsion. Size (**A**), PDI (**B**), pH (**C**), Zeta potential (**D**), and electrical conductivity (**E**). Same lowercase (a) letters indicate no significant statistical difference (*p* < 0.05) by One-way test.

In addition, the nanoemulsion showed pH 7.8 and a zeta potential value of −21.9 mV. The electrical conductivity was 0.38 mS/cm and this value was maintained in the subsequent measurements. It is worth highlighting that all the parameters previously described were also evaluated during the 14 days after preparation of the formulation, and there were no changes as observed in Figure 3.

**Figure 4:**
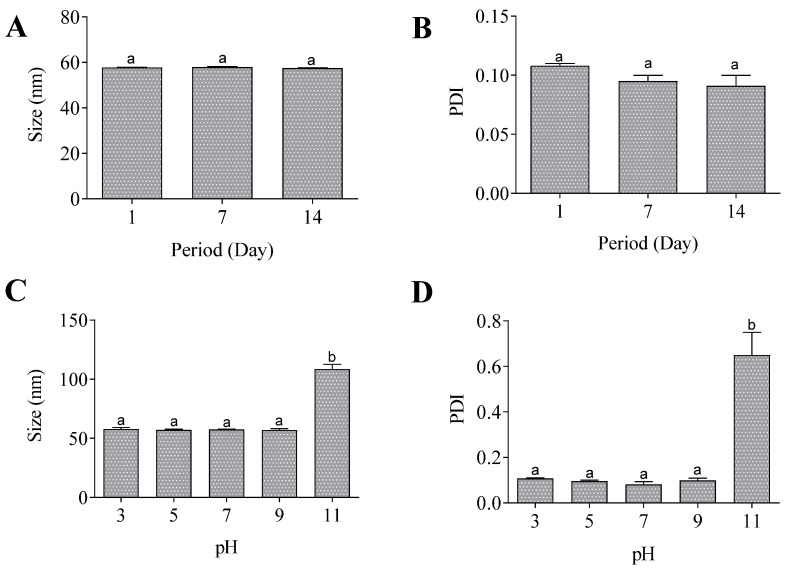
Accelerated stability of nanoemulsion. Size (**A**) and PDI (**B**) after centrifugation. Size (**C**) and PDI (**D**) after pH alterations. Same lowercase letters (a) indicate no significant statistical difference and different lowercase letters (b) indicates statistical difference (*p* < 0.05) by One-way test.

According to the accelerated stability assays, the nanoemulsion was stable after being subjected to high speed since no change in the mean particle size and PDI was observed (Figure 4A,B). Additionally, the nanoemulsion exhibited stability across a pH range of 3 to 9, with an augmentation in size and PDI solely at pH 11 (Figure 4C,D).

**Figure 5:**
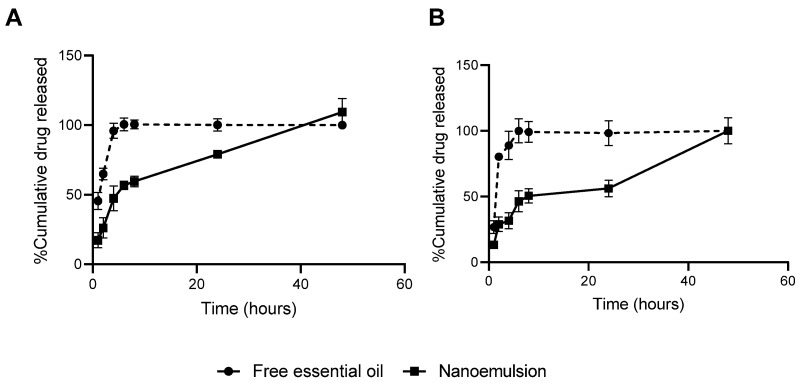
In vitro release of free essential oil and nanoemulsion expressed as percentage of cumulative drug released in pH 2.5 (**A**) and 7.5 (**B**).

The release profile of the essential oil from the nanoemulsion was found to be analogous across the evaluated pH values (pH 2.5 and 7.5). The developed nanoemulsion can be regarded as a sustained-release formulation, as it demonstrated maximum release within 48 h and free oil within 8 h (Figure 5).

Table 2 presents the adjusted kinetic coefficient (R^2^) data.

The release kinetics of the essential oil-loaded nanoemulsion were best described by the Higuchi model, with coefficients of determination of R^2^ = 0.9184 at pH 7.5 and R^2^ = 0.9453 at pH 2.5, indicating that passive diffusion driven by concentration gradient is the predominant mechanism under both conditions. However, the Korsmeyer–Peppas model exhibited a robust fit as well (R^2^ = 0.9067 at pH 7.5 and R^2^ = 0.9385 at pH 2.5), with values closely approximating those of the Higuchi model. This similarity suggests the contribution of additional release mechanisms [28].

In order to provide a more thorough elucidation of the release dynamics, the parameters *n* and k from the Korsmeyer–Peppas model were analyzed. The release exponent *n* exceeded 2 in both conditions (*n* = 2.0311 at pH 7.5; *n* = 2.0576 at pH 2.5), which is characteristic of super case II transport. This transport is typically associated with complex structural changes such as matrix relaxation and controlled erosion, in addition to diffusion. Furthermore, the higher release rate constant observed at pH 2.5 (k = 0.8034) compared to pH 7.5 (k = 0.6117) supports the hypothesis of an accelerated release under acidic conditions, potentially due to colloidal destabilization or increased solubility of the essential oil in the receptor medium at low pH [29].

### 3.3. Antibacterial Activity

In the screening of microorganisms susceptible to the *M. graveolens* essential oil, growth inhibition zone was observed for all the Gram-positive bacteria evaluated (Table 2). On the other hand, all Gram-negative bacteria and yeast were resistant to the essential oil.

The antimicrobial potential of the essential oil was also evaluated quantitatively for the susceptible microorganisms and MIC values ranging from 250.0 to 15.6 mg/mL are shown in Table 2. In addition, the nanoemulsion was able to reproduce this potential with lower MIC values (from 25.0 to 3.12 mg/mL) and the control formulation showed no effect against the microorganism (Table 3).

### 3.4. Trypanocidal Activity

The action of the *M. graveolens* against *T. cruzi* was evaluated and IC50 values of 2.3 and 60.4 mg/mL were found for the free and encapsulated essential oil, respectively (Table 4).

### 3.5. Cytotoxicity Assay

The cytotoxicity result is reported in Table 3 and CC_50_ values of 0.62 and 9.5 mg/mL were found for the free and encapsulated essential oil, respectively.

## 4. Discussion

The *M. graveolens* essential oil showed a viscous aspect and a delightful smell that could have commercial value as fragrance for cosmetics or home care products, similar to what has been reported by Toudahl et al. [3]. The yield of oil extraction was consistent with climatic characteristics, since the lowest oil content was generally obtained during the dry season due to the low vegetative growth and the absence of flowers. On the other hand, the oil production increased in the months with the highest proportions of rain (November to March) [9,21,30]. A comparison of the present study’s yield (1.6% and 1.9%) with those of other studies reveals that it is lower than the yield reported by Toudahl et al. [3]. In their study, Toudahl et al., collected this species in October in the same region and found a markedly superior yield (4.8%) [3]. However, the present yield was higher than the values reported by Pereira et al. [9] for the dry season (yield 0.9%) and rainy season (yield 0.7%) essential oil extraction of *M. crenulata*. It is suggested that this variation may occur due to the lower moisture content of the leaves in dry weather and a higher yield when compared to the mass of botanical material used for oil extraction.

The chemical similarity of both oils suggests the homogeneity of its constitution independent of the collection period and are important to ensure the product quality from a natural product. It is also worth highlighting the high amount of *cis*-pinocarvyl acetate in the *M. graveolens* essential oil that would allow a high yield and easy obtaining of this metabolite. On the other hand, studies performed by Toudahl et al. [3] showed trans-pinocarvil acetate (78.9%) and β-pinene (3.8%) as main components of this oil. This difference may have occurred due to the similarity of the mass spectra and the difficulty in identifying the compounds, since *cis* and *trans*-pinocarvil acetate are stereoisomer and only geometric conformation is distinct. However, this problem can be minimized by calculating KI [31].

The nanoemulsion obtained showed mean particle size in agreement with the definition that o/w dispersions with particle size ranging between 50 and 500 nm are considered nanoemulsions [32]. PDI refers to particle diameter distribution and classify the formulation as monodispersed (0) or polydispersed (1). A lower PDI value indicates greater homogeneity in the system [33]. PDI of 0.09 was found in our work and shows uniformity of the particle population.

Nanoemulsions loaded with different essential oils have already been obtained by other authors. According to Wan et al. [34], formulations containing essential oils from thyme, lemongrass, cinnamon, peppermint, and cloves showed particle size smaller than 200 nm. Values of particle size (77 nm) and PDI (0.09) close to our results were found by Seibert et al. [21] after encapsulation of *C. densiflorus* essential oil. Similarly, size of 79 nm and monodisperse distribution are characteristics of the nanoemulsified *C. flexuosos* oil [33]. In addition, a uniform population and size lower than 200 nm was found for the nanoemulsions containing oil from *Carapa guaianiensis* and *Schimus molle* [35].

Another factor commonly used to monitor the formulation stability is the hydrogen potential (pH), since changes in its value suggest the occurrence of chemical reactions. In the present study, the pH 7.8 may be related to the constituents of the formulation since the one without essential oil showed similar pH (pH 7.9).

Zeta potential was also evaluated since the electrostatic repulsion between the suspended particles can prevent the aggregation process and, consequently, improve the stability of the system [36,37]. Although values below |30| mV for this parameter are indicative of instability, it is possible that the colloidal dispersions remain stable due to the steric effect provided by the bulky surface groups of the nonionic surfactants [38]. This process would justify the stability of the developed nanoemulsion, even with a zeta potential value of −21.9 mV. In addition, the negative value may be related to the absorption of anionic species from the water to the surface of the drops or anionic impurities when nonionic surfactants are used [39]. Other studies have also produced stable nanoemulsions with low zeta potential values [21,33,40,41].

In addition, the assessment of electrical conductivity can be employed as a method for evaluating the stability of o/w systems, since higher values are observed when water is the continuous phase. In this case, reduced values refer to exposure of oily droplet and formulation instability [21]. All evaluated parameters remained unchanged for a period of 14 days, suggesting the production of stable nanoemulsion.

In addition to evaluating stability in storage at room temperature, an accelerated stability test was conducted, since it is an important strategy to predict the quality of a product. These assays accelerate the appearance of possible instability processes that can occur during storage for long periods. The centrifugation test can favor the separation of phases due to the creaming or coalescence processes [42]. The nanoemulsion was stable after being subjected to high speed with similar mean particle size and PDI. Similarly, these parameters were maintained in a nanoemulsion containing propolis extract after thermal stress and centrifugation assays [22]. These methods were also not enough to destabilize the nanostructured system loaded with *C. densiflorus* essential oil [21].

Furthermore, the stability of the formulation was verified by measuring pH changes between 3 and 9. These findings suggest that the formulation’s characteristics are sustained in the gastrointestinal tract following oral administration. Of the potential routes of administration, the oral route is of particular significance in terms of patient compliance with prescribed drug regimens [43]. The capacity of nanoemulsions to augment the efficacy of oral medications has been substantiated by studies that documented substantial enhancements in bioavailability, stability, and absorption, particularly for drugs and bioactive compounds that are characterized by poor solubility [44].

Another advantage of oral nanoemulsions is the possibility of prolonged release, which was observed in the in vitro testing of the nanoemulsion developed here at pH levels equivalent to those of the stomach and small intestine. The sustained release of the drug has the potential to optimize its pharmacokinetics, thereby reducing toxicity and minimizing off-site adverse effects [44].

In the screening of antimicrobial activity, only Gram-positive bacteria were susceptible to the *M. graveolens* essential oil, and this report is the first to demonstrate the bactericidal effect of *M. graveolens*. Studies performed by Pereira et al. [9] and Cassiano et al. [8] have already shown the antimicrobial activity for *M. cremulata* and *M. hatschbachii*, respectively. However, the last species was also active against Gram-negative bacteria and this difference may be related to the sample evaluated and extraction method [8]. Cassiano et al., obtained the extract from the dried aerial parts of *M. hatschbachii* by maceration with hexane, followed by removal of the solvent [8]. The authors reported the composition of this extract, identifying the presence of hydrocarbons, methyl esters of long-chain fatty acids, linear carboxylic acids, and diterpenes [8]. In addition to the species being different, the differences in composition compared to the present study may also be due to the extraction method, which was performed by hydrodistillation. The hydrodistillation process is known to yield a predominant proportion of monoterpenes and sesquiterpenes, in contrast to hexane extraction, which predominantly results in the extraction of fatty acids and alkanes [45]. Opposite performance was also observed for the *M. crenulata* since the oil was capable of inhibiting the *P. aeruginosa* but it was not active against Gram-positive strains evaluated. On the other hand, the result of resistance of *Candida* genus to this species is in agreement with our work [17].

Although the essential oil evaluated was the same in all tests, the relationship between the size of the inhibition zone obtained by the agar diffusion method and the MIC values determined by serial dilution was not directly proportional among the different microorganisms tested. This lack of correlation can be attributed to multiple factors, primarily structural and physiological differences between the microorganisms. The diffusion of the essential oil in solid media depends on its physicochemical properties, such as volatility and hydrophobicity, as well as its interaction with the agar components. However, the antimicrobial efficacy determined by the MIC in liquid media is related to the direct and prolonged contact of the compound with the microorganism. Therefore, the combined use of diffusion and dilution methods is essential for a more complete assessment of antimicrobial activity, especially in the case of complex substances such as essential oils. The lack of correlation between inhibition zone and MIC has already been demonstrated in other studies with essential oils [46,47].

The nanoemulsion was able to reproduce the antibacterial effect of the *M. graveolens* essential oil. These results are important to enable the use of essential oils since nanostructured systems are more stable and have shown enhanced biological activities of the encapsulated lipophilic component due to their reduced particle size and increased solubility [48]. In addition to enhanced solubility, the elevated activity of nanoemulsions in comparison to the free essential oil may be ascribed to the potential for penetration into bacterial cells by means of fusion with the lipid bilayer interface with the cell wall, thereby facilitating intracellular drug delivery [49].

In this way, studies showed that nanoemulsions containing *C. flexuosus* essential oil exhibited significant antimicrobial properties, with values of minimum inhibitory concentration lower than those presented by free oil [33,41]. The same results were also demonstrated for nanoemulsified essential oil from *C. densiflorus* [23]. Even at the highest concentration, the active-free nanoemulsion (NE-NC) did not inhibit bacterial growth. This result may be due to the low percentage of surfactants (10% total) in the nanoemulsion composition and the lack of antimicrobial activity of corn oil at the tested concentrations.

*Staphylococcus* species were the most susceptible to free and nanoemulsified essential oil. However, it is worth highlighting that nanoemulsion enhanced the essential oil effect on *L. monocytogenes* by more than 80 times. Species from both genera are examples of pathogenic bacteria and may cause diverse infections in humans. In addition, they are important targets for research due to the growing appearance of resistant strains [50,51]. Thus, these data demonstrate the potential use of this new product to combat diseases caused by these microorganisms.

In relation to the trypanocidal activity, this is the first report about the action of the *M. graveolens* against *T. cruzi*, but a previous study has already demonstrated antiprotozoal activity of *M. crenulata* [7]. According to the author, the most apolar fractions, dichlorometane and hexane, were active against *Plasmodium falciparum*. However, the ability to inhibit parasitic growth was reported for the first time for *M. graveolens*. In agreement with our result, *Cinnamodendron dinisii* also showed anti-*Trypanosoma cruzi* activity (IC_50_ 283 µg/mL) [52]. The essential oil extracted from this species is mainly composed of α-pinene (36%), β-pinene (18%), and sabinene (12%), suggesting that these compounds may be related to the trypanocidal action.

Although nanoemulsion did not enhance the effect of *M. graveolens* essential oil, other data prove the efficiency of this system. A significant reduction in the number of *Trypanosoma* forms was observed after treatment with nanoemulsions containing essential oil from *Carapa guaianiensis* and *Schimus molle* [35]. In addition, nanoemulsified essential oil from *Pterodon emarginatus* showed antiparasitic activity against *Anacanthorus spathulatus*, *Notozothecium janauachensis*, and *Mymarothecium boegeri* [53].

The cytotoxicity was also evaluated, and although this is the first report about *M. graveolens* cytotoxicity, the results show its safety when compared to other species. According to Pereira et al. [7], dichloromethane fraction of *M. crenulata* showed low effect on HepG2 cells growth (CC_50_ 220.56 µg/mL). In the screening of essential oils cytotoxicity on Vero cells, CC_50_ values ranging from 50 to 280 µg/mL were found for the most and least toxic species, respectively [1]. In addition, nanoemulsion reduced this effect by more than 15 times. This result may be related to characteristics of nanostructured systems, such as controlled drug release and, consequently, increased action time and decreased drug concentration peaks [54].

## 5. Conclusions

*M. graveolens* essential oil showed high proportions of the *cis*-pinocarvyl acetate, independent of the collection period. The antibacterial and trypanocidal activities were demonstrated for the first time for this oil. Free and nanoemulsified oil showed high potential against Gram-positive strains and the formulation reduced MIC values by up to 80 times. In addition, reduced cytotoxicity was found after encapsulation of this essential oil. Thus, the stable developed nanoemulsion is a great alternative to facilitate the use of bactericidal property of pinocarvyl acetate.

## Figures and Tables

**Table 1:** Chemical characterization of the *Microlicia graveolens* essential oil according to collection.

KI ^a^	Compound	Percentage (%)	
		Collection (1)	Collection (2)
939	α-pinene	0.85	0.67
975	Sabinene	0.31	0.33
979	β-pinene	6.70	5.66
990	Myrcene	2.94	2.68
1026	o-cymene	-	0.06
1029	Limonene	1.25	1.52
1031	1,8-cineol	0.88	-
1059	γ-terpinene	-	0.02
1164	Pinocarvone	-	0.38
1170	ρ-mentha-1,5-dien-8-ol	-	0.08
1175	*cis*-pinocamphone	-	0.18
1177	Terpinen-4-ol	-	0.10
1184	*cis*-pinocarveol	0.53	0.77
1188	α-terpineol	0.46	0.36
1195	Myrtenal	-	0.23
1200	*cis*-4-caranone	-	0.03
1282	*cis*-verbenyl acetate	-	0.17
1290	*trans*-sabinyl acetate	-	0.07
1298	*trans*-pinocarvyl acetate	1.66	3.39
1299	*cis*-α-necrodol acetate	-	0.29
1312	*cis*-pinocarvyl acetate	83.57	76.89
1326	Myrtenyl acetate	-	0.31
1342	*trans*-carvyl acetate	-	0.11
1376	α-copaene	-	0.26
1388	β-bourbonene	-	0.18
1419	*trans*-caryophyllene	-	0.54
1454	α-humulene	-	0.17
1479	γ-muurolene	-	0.22
1481	Germacrene D	-	1.08
1496	2-tridecanone	-	0.06
1500	Bicyclogermacrene	-	0.32
1505	β-Bisabolene	-	0.37
1523	δ-cadinene	-	0.18
1531	*trans*-γ-bisabolene	-	0.06
1563	*trans*-nerolidol	-	0.17
1578	Spathulenol	-	0.18
1583	Caryophyllene oxide	-	0.09
1592	Viridiflorol	0.86	-
1760	Benzyl benzoate	-	0.26
1949	Pimaradiene	-	0.18
**Hydrocarbon monoterpene**		12.05	10.94
**Oxygenated monoterpene**		87.10	83.36
**Hydrocarbon sesquiterpene**		-	3.38
**Oxygenated sesquiterpene**		0.86	0.76
**Hydrocarbon diterpene**		-	0.18
**Total identified**		100.01	98.62

^a^ KI = Kovats index.

**Table 2:** Adjusted kinetic coefficient (R^2^) obtained by zero order, first order, Higuchi model, and Korsmeyer–Peppas model.

pH	Kinetic Models (R^2^)			
	Zero Order	First Order	Higuchi	Korsmeyer–Peppas
7.5	0.8869	0.6504	0.9184	0.9067
2.5	0.8520	0.6127	0.9453	0.9385

**Table 3:** Antibacterial activity of the free and nanoemulsified essential oil obtained from *Microlicia graveolens*.

Microorganism	IZ (mm)		MIC (mg/mL) *		
	EO	CNT	EO	NE	NE-NC
*Enterococcus faecalis*	16.5 ± 0.5	25.3 ± 1.4	250.0	25.0	Inactive
*Enterococcus faecium*	9.0 ± 0.0	19.8 ± 1.3	250.0	25.0	Inactive
*Listeria monocytogenes*	20.0 ± 0.0	27.6 ± 0.9	250.0	3.12	Inactive
*Staphylococcus aureus*	9.0 ± 0.0	21.9 ± 1.9	62.5	6.25	Inactive
*Staphylococcus saprophyticus*	12.0 ± 1.0	23.8 ± 0.7	15.6	3.12	Inactive

IZ—inhibition zone; MIC—minimal inhibition concentration; EO—free essential oil; NE—nanoemulsified essential oil; NE-NC—nanoemuslion negative control; CNT—positive control: tetracycline (100.0 µg/mL). * In the MIC test, no microbial growth was observed in the positive controls at the concentrations that were tested.

**Table 4:** Trypanocidal activity and cytotoxicity on L929 cells of the free and nanoemulsified essential oil obtained from *Microlicia graveolens*.

Sample	IC_50_ on Parasite ^a^ (mg/mL)	CC_50_ on L929 ^b^ (mg/mL)
**EO**	2.3	0.62
**NE**	60.4	9.5
**NE-NC**	Inactive	11.4

^a^ Concentration which reduced 50% of the proliferation of parasite cells. ^b^ Cytotoxic concentration for 50% of L929 cells. EO—free essential oil; NE—nanoemulsified essential oil; NE-NC—nanoemuslion negative control.

## Data Availability

Data is contained within the article.
